# Systemic immune-inflammation index could be associated with pseudophakic cystoid macular edema after an uneventful phacoemulsification surgery in patients without risk factors

**DOI:** 10.1186/s12886-022-02606-5

**Published:** 2022-09-21

**Authors:** Sücattin İlker Kocamış, Ali Altan Ertan Boz, İbrahim Özdemir

**Affiliations:** 1 Department of Ophthalmology, University of Health Sciences, Dışkapı Yıldırım Beyazıt Training and Research Hospital, Ziraat Mah. Şehit Ömer Halisdemir Cad. Dışkapı, Ankara, Turkey; 2 Department of Ophthalmology, Sakarya University, Sakarya Training and Research Hospital, Sakarya, Turkey; 3 Department of Ophthalmology, Dünyagöz Hospital, Sakarya, Turkey

**Keywords:** Pseudophakic cystoid macular edema, Systemic immune-inflammation index, Serum biomarkers, Neutrophil-to-lymphocyte ratio, Platelet-to-lymphocyte ratio

## Abstract

**Background:**

To evaluate the association between serum biomarkers and pseudophakic cystoid macular edema (PCME) in eyes without risk factors after uneventful phacoemulsification cataract surgery.

**Methods:**

This is a case–control study. Patients without risk factors and who developed clinically significant PCME after uncomplicated phacoemulsification surgery were enrolled in the study. The age- and sex-matched control group that had normal fundus examination findings and 10/10 visual acuity in the first week, first month and following postoperative control visits was randomly recruited from the same study cohort. The neutrophil-to-lymphocyte ratio (NLR), platelet-to-lymphocyte ratio (PLR), and systemic immune-inflammation index (SII) were obtained from the preoperative complete blood count (CBC) test and compared between the two groups. Linear regression analysis was used to assess the relationship between central macular thickness (CMT) and biomarkers. A binary logistic regression model was generated to evaluate the significance of the biomarkers in predicting PCME. The receiver operating characteristic (ROC) curves of the significant parameters in the logistic regression model were presented to detect the area under the curve (AUC), the cut-off point, the sensitivity and the specificity.

**Results:**

The study cohort included 5352 patients. Of these patients, 52 (0.97%) met the inclusion criteria, and 60 age- and sex-matched patients were recruited as the control group. PLR, NLR, and SII were significantly different between the two groups (*p* = 0.006, *p* = 0.002, *p* < 0.001, respectively). According to the linear regression analysis, SII was found to have a significant relationship with CMT (*p* < 0.001). Only SII was assessed as significant in the logistic regression model (*p* = 0.046). In the ROC curve, the AUC of SII was 0.709. The sensitivity and specificity of SII for PCME prediction were 65.38% and 75%, respectively, and the cut-off point was 433.70.

**Conclusion:**

SII is associated with the occurrence of PCME in eyes without risk factors after uneventful phacoemulsification surgery. SII could be a useful tool to predict PCME in eyes without risk factors.

## Background

Pseudophakic cystoid macular edema (PCME) is the most significant cause of unexpected visual loss after phacoemulsification cataract surgery [1]. Although systemic diseases such as diabetes and hypertension, ocular conditions such as epiretinal membrane and retinal vein occlusion, and surgical complications such as posterior capsule rupture and vitreous loss are risk factors for the development of PCME [2], it is not uncommon for PCME to occur after an uncomplicated surgery without risk factors [3].

Although the exact pathophysiology of PCME remains unknown, inflammatory processes that are activated and accelerated by surgery are thought to play an important role in PCME formation [4]. Therefore, nonsteroidal anti-inflammatory (NSAID) drugs were administered for prophylaxis and treatment of PCME to inhibit prostaglandin production and its subsequent inflammatory effects [5]. However, some studies have suggested that NSAIDs showed no or negligible benefit in patients without risk factors [6, 7]. Therefore, it is important to investigate the etiology of PCME in more detail.

Since peripheral blood biomarkers are inexpensive, easily accessible and common measurements, they have recently been the focus of research. Studies have suggested that they could be potential candidates as pro-inflammatory and pro-angiogenic biomarkers for various systemic diseases and morbidity [8–13]. They were also associated with some ocular disorders such as diabetic retinopathy, central serous chorioretinopathy, wet age-related macular degeneration, and retinal vein occlusion as inflammatory markers [14–16]. Accordingly, this study aimed to evaluate the possible relationship between peripheral blood biomarkers and PCME in patients without risk factors after uneventful cataract surgery.

## Methods

This study was approved by the Ethics Committee of Sakarya University Medical School (No: 02.02.2022–102,085) and was conducted in accordance with the tenets of the Declaration of Helsinki. The need for written informed consent was waived by the Sakarya University Medical School Ethics Committee due to retrospective nature of the study.

The medical records of patients who underwent phacoemulsification surgery between June 2015 and January 2022 by the same three experienced surgeons (AAEB, SIK, and İÖ) at the Sakarya University Sakarya Training and Research Hospital and Sakarya Yenikent State Hospital were retrospectively evaluated.

**Fig. 1:**
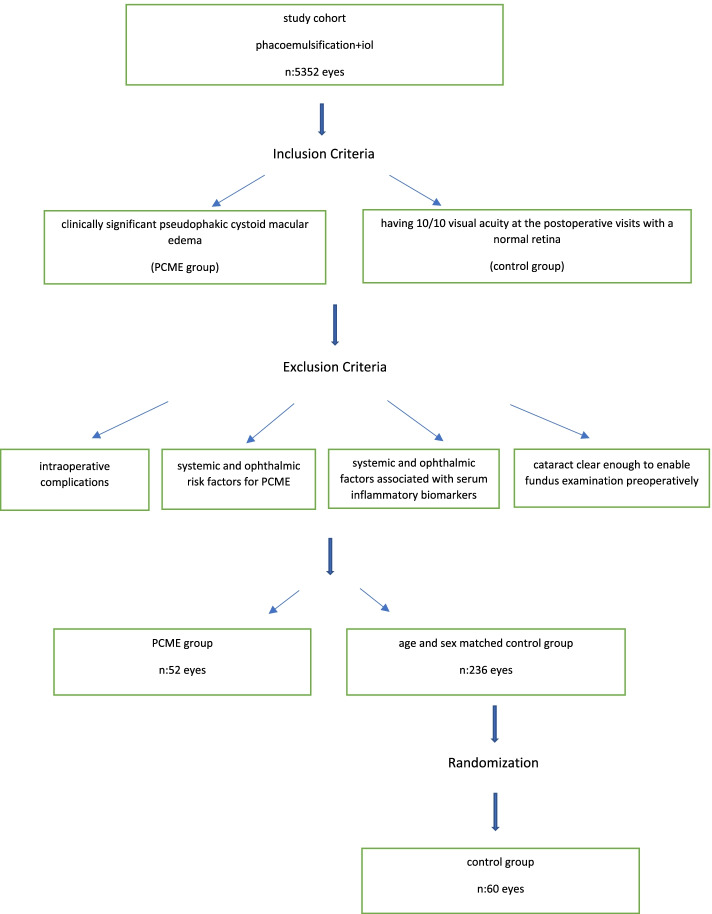
Flowchart showing the study design

Patients who developed clinically significant PCME after an uncomplicated phacoemulsification surgery were enrolled in the study. All surgeries were performed with Infiniti with Ozil and IP system and Centurion Vision System (Alcon, Fort Worth, TX, USA). Clinically significant PCME was defined as retinal cysts located at the internal nuclear layer and outer plexiform layer confirmed with optical coherence tomography (Cirrus HD-OCT 4000, Zeiss, Jena, Germany) along with the decrease in at least two lines in the expected best corrected visual acuity. Patients who had systemic diseases such as diabetes mellitus, hypertension, malignancy, cardiovascular diseases, systemic infections, and inflammatory diseases were not included. Patients taking any immunosuppressive drugs were also not included in the study. The exclusion criteria for the ocular conditions were previous ocular surgery in the same eye, history of PCME in the contralateral eye, history of uveitis, glaucoma, and use of glaucoma medication, pseudoexfoliation syndrome, any sign of retinal disease on the fundus examination, such as epiretinal membrane, age-related macular degeneration, retinal vein occlusion, degenerative myopia, and cataracts dense enough to prevent fundus examination. The exclusion criteria for surgical complications were posterior capsule rupture, vitreous loss or incarceration, iris trauma, retained nucleus fragments, iris, and sulcus fixated intraocular lens. The age- and sex-matched control group was recruited from the same study cohort with the same inclusion and exclusion criteria, except for having a normal fundus examination and a 10/10 visual acuity in the first week, first month, and following postoperative control visits. There were 236 eyes which were fitting the selection criteria for the control group. Then a computer program was used to select 60 eyes to achieve randomization. Figure 1 summarizes the study design.

The patients underwent a detailed ophthalmological examination, including visual acuity measurement, anterior segment and dilated fundus evaluation with a biomicroscope, intraocular pressure measurements, optical biometry measurement, optical coherence tomography, and fundus fluorescein angiography if needed preoperatively. Postoperatively, steroid and antibiotic eye drops were started eight times a day and gradually decreased for 1 month. Routine control examinations were performed 1 day, 1 week and 1 month after the operation if abnormalities were not detected.

Complete blood count (CBC) testing was routinely requested from all patients prior to surgery. After 8–12 h of fasting, blood samples were collected from the antecubital vein and analyzed with the BC-6800 hematology analyzer (mindray, Shenzhen, Nanshan, PR China) 2 h after collection. Neutrophil, lymphocyte, platelet counts, mean platelet volume (MPV), and red cell distribution width (RDW) were obtained from CBC testing. The neutrophil-to-lymphocyte ratio (NLR) was calculated by dividing the neutrophil count by the lymphocyte count (neutrophil/lymphocyte), the platelet-to-lymphocyte ratio (PLR) was calculated by dividing the platelet count by the lymphocyte count (neutrophil/lymphocyte), and the systemic immune-inflammation index (SII) was calculated as follows: (neutrophil × platelet)/lymphocyte.

After checking the distribution of the variables using the Shapiro–Wilk test, the means of the variables were compared between the two groups using the Chi-square test, independent samples t test, and Mann–Whitney U test. Linear regression analysis was used to assess the relationship between central macular thickness (CMT) and the biomarkers found to be significant in the independent samples t-test. A binary logistic regression model was generated to evaluate the significance of the same parameters in predicting PCME. The receiver operating characteristic (ROC) curves of the significant parameters in the logistic regression model were presented to detect the area under the curve (AUC), the cut-off point, the sensitivity and the specificity of these parameters at the identified cut-off point. A *p*-value < 0.05 was accepted as significant. Jamovi version 2.2.5 was used for the statistical analysis.

## Results

A total of 5352 patients were included in the study cohort. Of these patients, 52 (0.97%) met the inclusion criteria, and 60 age- and sex-matched patients were randomly recruited as the control group. The descriptive statistics of the groups are detailed in Table 1. The mean postoperative PCME diagnosis time was 38.8 ± 10.4 (30–75) days. The mean CMT of the PCME group at the time of diagnosis was 366 ± 34.6 (314–439) µm.

**Table 1:** Descriptive statistics of the groups

	PCME	Control	*P*-value
Gender	30 M, 22 F	35 M, 25 F	0.945*
Age (mean, Std, range)	66.1 ± 4.89 (57–74)	65.5 ± 4.10 (55–73)	0.488**
Preop VA (logMAR) (median, range)	0.222 (0.155–0.398)	0.301 (0.155–0.398)	0.303***
Preop IOP (mean, Std, range)	16 ± 2.04 mmHg (12–20)	15.6 ± 1.76 mmHg (12–20)	0.254**
Device	35 Infiniti, 17 Centurion	37 Infiniti, 23 Centurion	0.534*
Surgeon	SIK 19, AAEB 21, İÖ 12	SIK 21, AAEB 24, İÖ 15	0.969*

*PCME* pseudophakic cystoid macular edema, *Std* standard deviation, *Preop VA* preoperative visual acuity, *Preop IOP* preoperative intraocular pressure

^*^Chi-square test

^**^Independent samples t-test

^***^Mann–Whitney U test

When the peripheral blood biomarkers and inflammatory parameters were compared, PLR, NLR, and SII were significantly different between the two groups (*p* = 0.006, *p* = 0.002, *p* < 0.001, respectively). Table 2 demonstrates the details of the comparison.

**Table 2:** Comparison of the peripheral blood biomarkers

	PCME	Control	*P*-value
Neutrophile (mean, Std, range)	3.54 ± 0.432 (2.56–4.27)	3.37 ± 0.547 (2.44–4.81)	0.069*
Lymphocyte (mean, Std, range)	2.30 ± 0.328 (1.83–3.12)	2.41 ± 0.341 (1.56–3.28)	0.097*
Platelet (mean, Std, range)	292 ± 39.3 (212–374)	280 ± 31.9 (230–354)	0.060*
RDW (mean, Std, range)	14.4 ± 0.629 (12.8–15.7)	14.2 ± 0.547 (12.8–15.2)	0.059*
MPV (mean, Std, range)	9.21 ± 0.511 (7.80–10.4)	9.22 ± 0.515 (8–10.3)	0.897*
PLR (mean, Std, range)	129 ± 21.5 (69.9–178)	118 ± 19.5 (82.7–179)	0.006*
NLR (mean, Std, range)	1.56 ± 0.247 (1.14–2.16)	1.41 ± 0.230 (0.881–1.92)	0.002*
SII (mean, Std, range)	455 ± 84.5 (284–596)	393 ± 65.7 (285–522)	< 0.001*

*PCME* pseudophakic cystoid Macular edema, *Std* standard deviation, *RDW* red cell distribution width, *MPV* mean platelet volume, *PLR* platelet to lymphocyte ratio, *NLR* neutrophil to lymphocyte ratio, *SII* systemic immune-inflammation index

^*^Independent samples t-test

As a result of linear regression analysis, SII was found to have a significant relationship with CMT (*R*^2^ = 0.455, *p* < 0.001). Table 3 shows the linear regression model and the collinearity statistics of the variables.

**Table 3:** Linear regression analysis model including NLR, PLR, and SII as predictors of CMT and the collinearity statistics of the variables

Predictor	Estimate	Standard Error	T value	*P* value	Collinearity statistics	
					VIF	Tolerance
Intercept	221.44	28.37	7.81	< 0.001		
PLR	-0.19	0.27	-0.70	0.49	2.53	0.39
SII	0.35	0.092	3.79	** < 0.001**	4.59	0.22
NLR	-20.81	22.98	-0.91	0.37	2.42	0.41

(Coefficient of determination of the model) *R*^2^ = 0.455

*PCME* pseudophakic cystoid Macular edema, *CMT* central macular thickness, *NLR* neutrophil to lymphocyte ratio, *PLR* platelet to lymphocyte ratio, *SII* systemic immune-inflammation index, *VIF* variance inflation factor

When binomial logistic regression analysis was performed to generate a model to predict the occurrence of PCME, only SII was considered significant (*p* = 0.046). The logistic regression model is demonstrated in Table 4.

**Table 4:** The binomial logistic regression model including NLR, PLR, and SII as predictors of PCME and the collinearity statistics of the variables

Predictor	Estimate	Standard Error	Z value	*P* value	Collinearity statistics	
					VIF	Tolerance
Intercept	4.69	1.63	2.89	0.005		
PLR	-5.01e-4	0.01	-0.04	0.97	1.79	0.56
SII	-0.01	0.01	-2.00	**0.046**	3.51	0.29
NLR	-0.05	1.35	-0.04	0.97	2.36	0.42

*PCME* pseudophakic cystoid macular edema, *PLR* platelet to lymphocyte ratio, *SII* systemic immune-inflammation index, *NLR*: neutrophil to lymphocyte ratio, *VIF* variance inflation factor

**Fig. 2:**
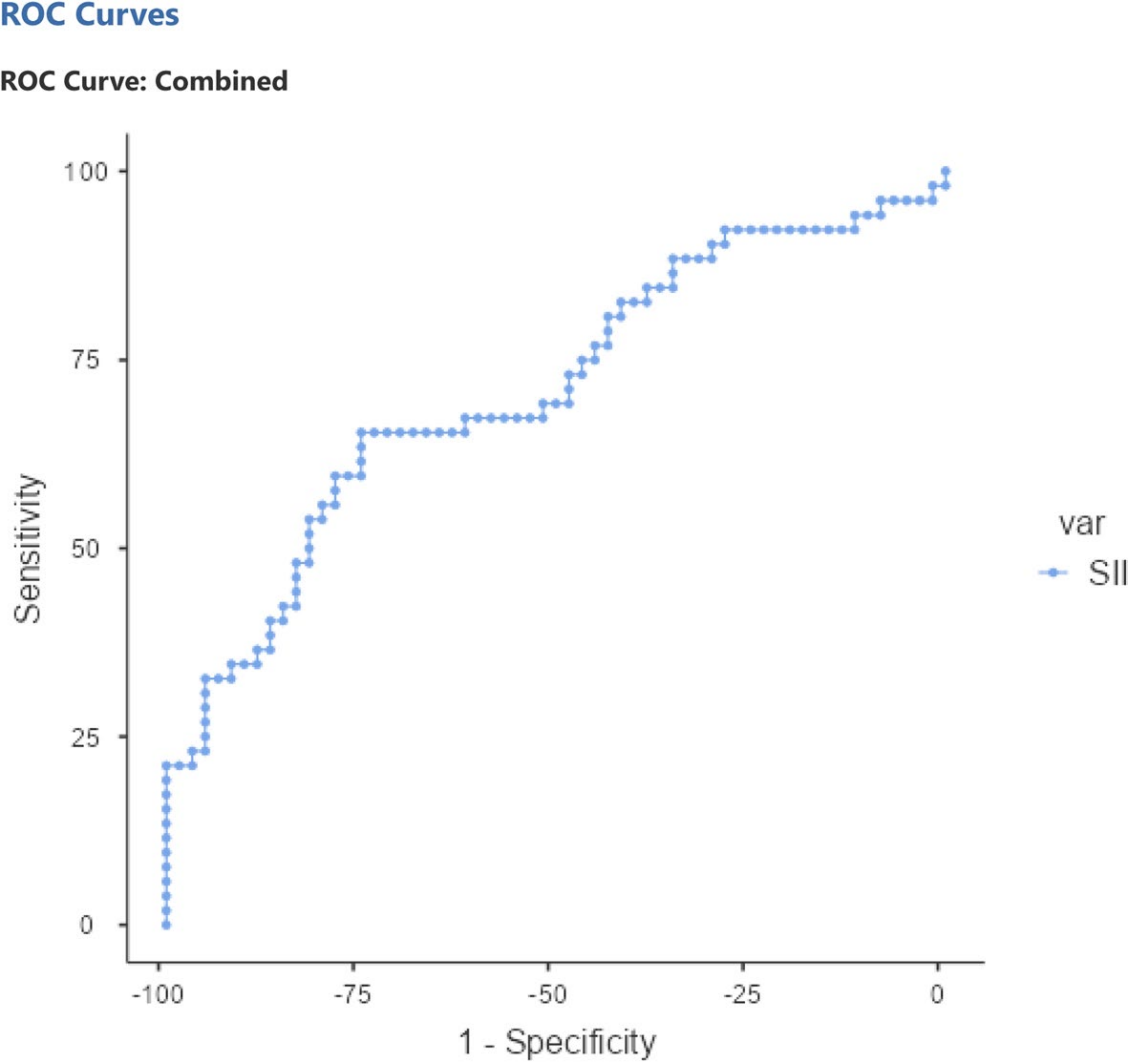
ROC curve of SII

According to the ROC curve, the AUC of SII was 0.709. The sensitivity and specificity of SII for PCME prediction were 65.38% and 75%, respectively, and the cut-off point was 433.70. Figure 2 represents the ROC curve of SII.

## Discussion

In this study, the incidence of clinically significant PCME after uncomplicated phacoemulsification surgery was 0.97% in eyes without risk factors. This rate was similar to the rate (1.17%) reported by Chu et al. [3], whose study on PCME incidence and risk factors is the largest to date. They found male sex and older age as risk factors. As we enrolled patients in an age- and sex-matched control group, our results could not have been affected by this aspect.

Our results showed that SII, NLR, and PLR were higher in the PCME group than in the control group, and among them, only SII was the predictor of PCME occurrence. To the best of our knowledge, this study is the first to investigate the relationship between CBC biomarkers and PCME. During inflammation, neutrophils and platelets are upregulated, whereas lymphocytes are downregulated [17, 18]. Thus, various inflammatory parameters, including these three cells derived from CBC, were introduced to investigate their availability as predictors or prognostic factors for many pathologies [19, 20].

PLR is one of these inflammatory parameters that have already been associated with some ocular diseases, such as glaucoma, uveitis, and retinal vein occlusion [21–23]. Since PLR is an indicator of both inflammation and thrombosis [24], it can be expected to identify a disease accompanied by vascular pathology. Blood–retinal barrier disruption and retinal capillary dilatation occur in the pathophysiology of PCME [25]. We found that PLR was significantly higher in PCME, but this significance was lesser than those of NLR and SII. MPV, a biomarker of platelet activity, was not different between the groups. Furthermore, RDW, an indicator of the susceptibility of erythrocytes to damage under oxidative stress [26], was not different between the groups. Thus, we could suggest that platelet activity, thrombosis, and erythrocyte heterogeneity might not participate significantly in PCME pathophysiology.

NLR represents both the pro-inflammatory activity of neutrophils and the immunomodulatory effects of lymphocytes; thus, it has the potential to strongly reflect the systemic inflammatory status. Many studies have shown that NLR is related to the occurrence and prognosis of many ophthalmic diseases [27, 28]. Although NLR was significantly higher in the PCME group than in the control group, it was not a predictor in the logistic regression analysis model in our study.

SII is a relatively new CBC biomarker and has the advantage of including all three, i.e., neutrophils, lymphocytes, and platelet counts, in its formula. Therefore, it may accurately reflect the immune-inflammatory state. The diagnostic and prognostic values of SII in some ophthalmological diseases were already presented [29, 30]. In our study, SII had the highest correlation with the CMT of the PCME group, and most importantly, SII was the only predictor of PCME occurrence in the logistic regression model.

Elbeyli et al. [31] indicated that SII is a superior marker to determine diabetic macular edema in diabetic retinopathy compared to NLR and PLR. Kurtul et al. [32] examined the usefulness of NLR, PLR, and SII in determining disease severity and activity in noninfectious uveitis. They demonstrated that SII was the most robust biomarker in identifying the severity of anterior uveitis, which was classified according to the anterior chamber cell intensity, and SII was the only significant biomarker in detecting the severity of posterior uveitis, which was classified according to the severity of vitreous haze, and finally, they concluded that SII was the most beneficial parameter compared with NLR and PLR in assessing disease severity. PCME has some features similar to those of uveitis in its pathophysiology. Ersoy et al. stated that there was much more aqueous flare in eyes with PCME than in eyes without PCME after cataract surgery [33]. A study suggested that inflammatory mediators concentrated in the aqueous humor after cataract surgery penetrate the vitreous by crossing the blood–aqueous barrier, and then damage the blood–retinal barrier [34]. Therefore, we suggest that SII could be an indicator of subclinical inflammation leading to the occurrence of PCME triggered by cataract surgery. Our results may shed light on research on the prophylaxis of PCME occurrence after cataract surgery. We think that systemic administration of NSAIDs prior to cataract surgery could be a focus of research for patients with high SII.

This study has some limitations. First, this study had a relatively small sample size. However, we had to meet strict inclusion and exclusion criteria for the appropriate study design. Second, detailed phacoemulsification parameters were lacking. However, all procedures were performed by surgeons with 10–15 years of phacoemulsification surgery experience, and all cataracts were clear enough to allow detailed fundus examination. Therefore, long surgery or aggressive phacoemulsification parameters would not be expected. Finally, there was a deficiency in other inflammatory parameters that can be obtained from the blood tests. In our clinical routine, we do not administer detailed blood tests other than CBC that include inflammatory parameters unless the patient has a remarkable medical history or an active disease.

## Conclusions

In conclusion, SII is associated with the occurrence of PCME in eyes without risk factors after uneventful phacoemulsification surgery. SII could be a useful tool to predict PCME in eyes without risk factors. Further studies with larger sample sizes involving more detailed inflammatory parameters are needed to better elucidate the possible relation between systemic subclinical inflammation and PCME.

## Data Availability

The datasets used and/or analyzed during the current study are available from the corresponding author on reasonable request.
